# Outcomes of distal femoral fractures treated with dynamic condylar screw (DCS) plate system: a single centre experience spanning 15 years

**DOI:** 10.11604/pamj.2021.38.363.27524

**Published:** 2021-04-14

**Authors:** Meryem Lemsanni, Youssef Najeb

**Affiliations:** 1 Department of Orthopaedic and Trauma Surgery, Ibn Tofail Hospital, Mohammed VI University Hospital Center, Abdelouahab Derraq Street, PB 40000, Marrakesh, Morocco

**Keywords:** Distal femur fracture, dynamic condylar screw, range of motion, Lysholm

## Abstract

**Introduction:**

the dynamic condylar screw (DCS) plate is an angular stable fixation (95°) system for distal femur fractures that allows for the ability to apply compression across the femoral condyles. The aim of this study was to evaluate our experience treating distal femur fractures with this device and give the long-term outcome.

**Methods:**

a retrospective study was undertaken in our institution during the period from January 2002 to December 2016. A total number of 240 patients with distal femur fractures were managed using DCS plate system and included in the study. The mean follow-up period was 33 months (26 - 62 months). Clinico-radiological progression of fracture union as well as the functional outcome was studied.

**Results:**

most of the fractures were closed injuries in young male patients resulting from traffic accidents. The average time to union was 12.6 weeks (range 11 - 23). Eight patients (3.3%) suffered superficial infection. Five patients (2.1%) developed deep venous thrombosis. Delayed union was observed in nine cases (3.7%) and non union in six cases (2.5%). At final follow-up, the mean range of motion (ROM) of knee was 115.7° (100°-148°). The mean Knee Society Score (KSS) was 84.5 (59 - 94) and the mean Lysholm score was 88.6 (range, 61 - 96), translating to good clinical results.

**Conclusion:**

our study conclusively establishes that excellent functional outcome can be achieved with DCS plate inserted with skilled surgical technique in distal femur fractures including the ones that are communited or occurring in osteoporotic bone with a negligible complication rates.

## Introduction

Around 3 to 6% of femoral fractures and less than 1% of all fractures occur to the distal part of the femur [1]. These injuries have a bi-modal distribution with the first peak being seen in the young resulting from high-energy trauma and the second peak being seen in the elderly osteoporotic population [2]. They are usually managed by open reduction and internal fixation. Achieving anatomical reconstruction by restoring articular congruity, limb alignment, length and rotation by using a rigid fixation method is essential for allowing early motion, adequate bone healing and avoiding future cartilage degeneration. Fractures in osteoporotic bone are particularly problematic due to poor bone stock for solid fixation as well as the propensity for intra-articular comminution [3]. The choice of implants and fixation techniques is made depending on the fracture pattern, degree of comminution, surgeon preference and patient choice. The range of options include screw fixation, fixed-angle devices (95° dynamic condylar screw (DCS) plates, 95° angle blade plate), pre-contoured locking plate, intramedullary nail, external fixation or total knee arthroplasty [4]. The aim of this study was to evaluate our experience treating distal femur fractures using DCS osteosynthesis and give the long-term outcome.

## Methods

We retrospectively reviewed 302 patients who underwent surgery for a distal femoral fracture at a tertiary care center between January 2002 and December 2016, for this institutional review board-approved study. The inclusion criteria were (1) age of at least 18 years, (2) presence of acute distal femoral fractures that were treated with osteosynthesis using DCS system, (3) and confirmed medical and radiological records. Patients with pathological fractures, previous knee injury, any fracture other than the distal femur in the ipsilateral limb were excluded. The parameters analyzed included age, gender, injured side, trauma mechanism, neurovascular status of the fractured limb, fracture patterns, soft tissue injuries, time to definitive treatment, postoperative rehabilitation, early and late complications. The fracture patterns were classified according to the AO/OTA Classification. Open fractures were classified using the method of Gustilo and Anderson.

### Surgical technique

The patients were given general or regional anesthesia at the discretion of the anesthesiologist. All of the surgeries were performed under tourniquet control in supine position on a radiolucent table. Intravenous administration of a first generation cephalosporin was given prophylactically. In open fractures, patients were taken to surgery on admission for thorough irrigation and debridement with excision of highly contaminated or necrotic soft tissue as well as non viable bone, in order to create an environment favourable to healing and to decrease infection risk. A first generation cephalosporin and an aminoglycoside were administered in the emergency room and continued for 5 days. A lateral approach to the distal femur was used, basic fracture principles were followed and the procedure was staged. First, the articular surface was reduced by clamping the medial and lateral condyles, then K-wires (one or two) were used to provide temporary stabilisation before being sequentially replaced with one to two 6.5 mm cancellous screws. After its reduction and fixation, the articular surface was brought to align with the metaphysis and temporarily stabilized with K-wires. Then, placement of the central guide wire in parallel to the knee joint axis was performed under image intensification. The DCS triple reamer was used to simultaneously drill for the lag screw, the plate barrel, and the plate/barrel junction. The DCS lag screw was inserted and the appropriate DCS plate was slided onto the guide shaft / lag screw assembly. Proximally, the plate was fixed to the femur by at least three bicortical 4.5 mm cortex screws. The wound was closed in layers and all patients had a Redon drain inserted, which was removed 48h post-operatively.

### Post-operative care and rehabilitation

Low molecular weight heparin was used in all patients until resumption of normal ambulation. Long leg splints were applied three weeks in all patients with severe comminution of fracture. Rehabilitation began two weeks after the operation including isometric quadriceps strengthening and passive range of motion. The weight-bearing time was defined according to the stability of fracture fixation and the healing situation of the limbs.

### Methods of assessment

The follow-up evaluation included clinical and radiographic assessments. All patients were followed up every month until the fracture union, and then annually until the most recent follow-up. The clinical functional assessment included the Knee Society Score (KSS), the Lysholm knee scoring scale, the range of motion (ROM) and the stability of the knee joint. The radiographic assessment included an evaluation of the fracture reduction, fracture union, and post-traumatic osteoarthritis. The union of the fracture was defined as the presence of a bridging callus in at least three cortices with no pain or tenderness over the fracture zone. The different union disorders, such as delayed union, nonunion, or malunion, were recorded in the study. Deep vein thrombosis, hardware failure, superficial or deep infection, peri-implant fracture were also noted.

### Statistical analysis

The collected data were analyzed using the Statistical Package of the Social Sciences (SPSS) version 25. Data were expressed as mean ± standard deviation, median, numbers, and percentages.

## Results

### Epidemiological characteristics

**Figure 1:**
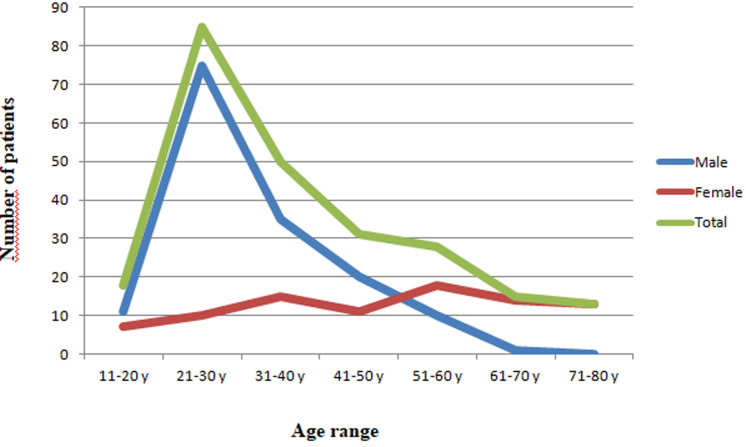
age distribution of the 240 fractures of the distal part of femur

Two hundred forty patients who fulfilled the inclusion criteria were enrolled in this study. The epidemiological characteristics and age distribution of the study cohort are shown in Table 1 and Figure 1, respectively. The mean age was 39.8 years (range 19 to 73). The overall age distribution has a peak at the third decade. The mechanism of injury is shown in Table 1.

**Table 1:** epidemiology of the 240 distal femoral fractures

	Number	%
**Gender**		
Male	152	63.3
Female	88	36.7
**Age**		
≤ 50	184	76.7
>50	56	23.3
**Injury mechanism**		
Traffic-related	151	62.9
Fall down from height	49	20.4
Simple fall	24	10
Sport-related	9	3.8
Miscellaneous	7	2.9
AO/OTA* Classification		
Type A:	90	37.5
A1	43	17.9
A3	47	19.6
Type C	150	62.5
C1	70	29.2
C2	45	18.75
C3	35	14.6
**Open fractures**	24	10
Type I (Gustilo and Anderson)	18	7.5
Type II (Gustilo and Anderson)	6	2.5

* AO/ OTA: Arbeitsgemeinschaft für Osteosynthesefragen/Orthopedic Trauma Association

### Clinical and radiological features

**Figure 2:**
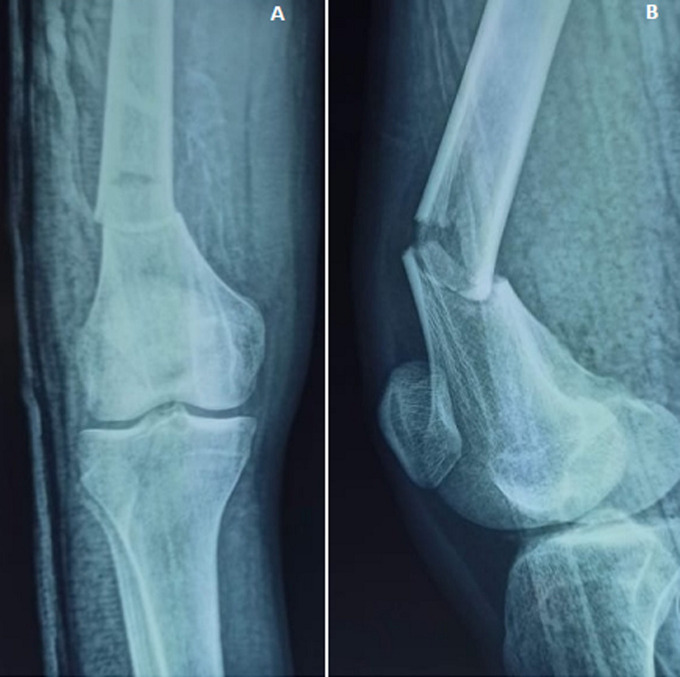
preoperative radiographs of the knee: anteroposterior (A) and lateral view (B); the images show distal femur fracture (A1 according to AO/OTA classification)

**Figure 3:**
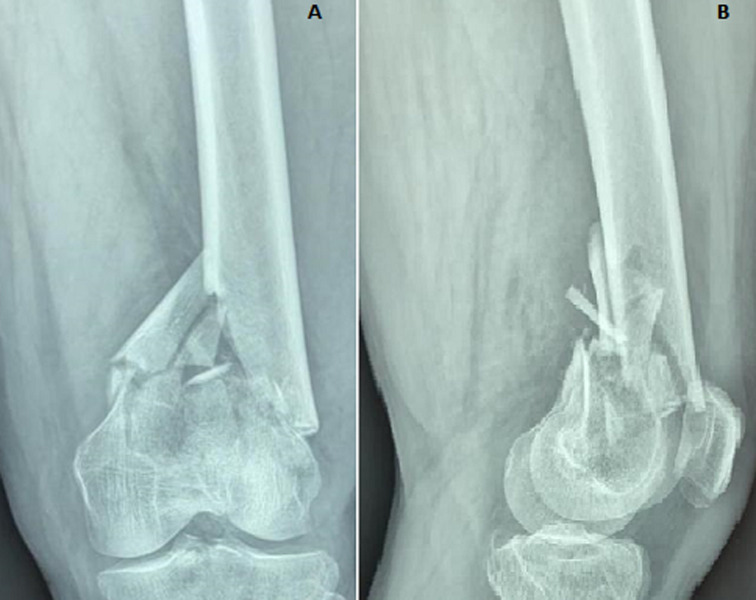
anteroposterior (A) and lateral (B) radiographs of a C2 type distal femoral fracture

**Figure 4:**
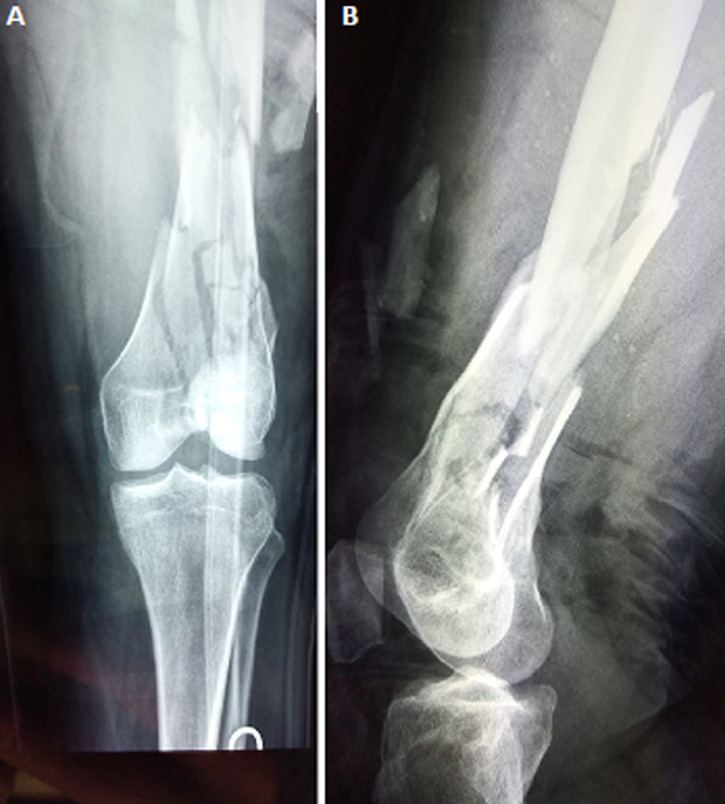
distal femoral fracture type C3: anteroposterior (A) and lateral (B) views of the knee

The right knee was affected in 58.3% (140/240) of cases and fractures were open in 24 patients (10%). In vascular examination, we found the dorsalis pedis and the posterior tibialis pulses were equal to the opposite leg. Neurological examination was performed and revealed no complications. According to the AO/OTA classification, and as shown in Table 1, 90 patients (37.5%) were type A (Figure 2) and 150 (62.5%) were type C (Figure 3, Figure 4).

### Surgical treatment

All fractures were managed with DCS plate system. The mean time from injury to surgery was about 10 hours (range, 6 - 17 h) for open fractures and 4.8 days (range, 2-7 days) in closed cases. Postoperative length of stay at hospital average was 5 days (range, 3 - 7 days).

### Complications

**Figure 5:**
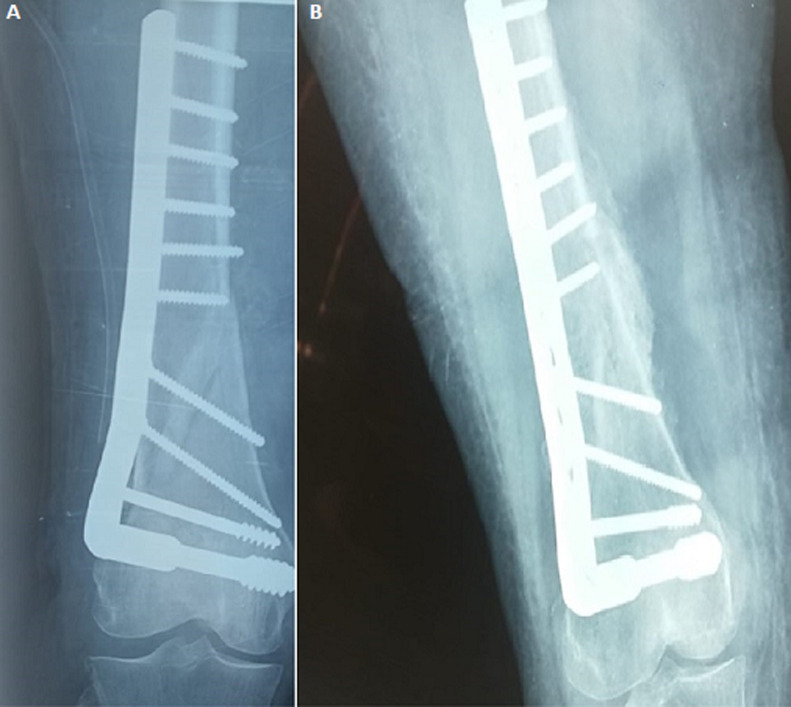
anteroposterior view X-rays showing good callus formation at end of four months (A) and six months (B) in an extra-articular distal femur fracture

Thirteen patients (5.4%) had early post-operative complications: there were eight cases (3.3%) of superficial infection that were successfully treated with culture sensitive parenteral antibiotics and antiseptic dressing; five patients (2.1%) had deep venous thrombosis and one of them developed pulmonary embolism, they required admission to the intensive care unit and anticoagulation therapy. All patients received follow-up, lasting for 26-62 months (average, 33 months). The average union time was 12.6 weeks with a range of 11 to 23 weeks (Figure 5). Delayed union was observed in nine cases (3.7%) and non union in six cases (2.5%). No internal fixation loosening or rupture, no loss of fracture reduction, no shortening and no varus/valgus instability was observed. Seven patients (2.9%) showed radiological evidence of secondary osteoarthritis of the knee joint. However, only three of these patients were symptomatic.

### Functional outcome

At final follow-up, the mean ROM of knee was 115.7° (100°-148°). The mean KSS was 84.5 (59-94). As per rating, 112 had excellent outcome, 109 had good, 17 had fair and 2 had poor outcome. The average Lysholm score was 88.6 (range, 61-96), with 119 excellent results, 93 good results, and 28 fair results (excellent/good rate: 88.3%).

## Discussion

Distal femur fractures are defined as fractures that affect the lower 9 to 15 centimetres of the femur, down to the articular surface of the knee [5]. The epidemiological features of the distal femoral fractures have been reported in European, Asian, Australian, American and African subjects [6-10], but they are not available in our region. Previous Studies show a bi-modal age distribution with a first peak in the third decade consisting mainly of men, and a second peak in the eighth decade, mostly women with osteoporotic fractures. Our result showed a single peak in the third decade, as most young males suffered from high-energy trauma. Their studies reflect the relatively lower incidence of high-energy trauma than in our region.

The optimal management of distal femoral fractures is still controversial. For several authors, the surgical treatment is the better choice in order to obtain a reduction and stable alignment and to prevent the complications [11]. There are different operating methods: plate osteosynthesis, intramedullary nails and external fixation. In the medical literature, there is a lack of important clinical studies that might guide the orthopaedic surgeon to reach a final decision as to which treatment to implement [12]. Nowadays, various studies consider plating superior to intramedullary nailing and it remains the preferred technique in the fractures of the distal femur [13]. While locked plating is widely prescribed in distal femoral fractures, DCS plating system is not frequently used because some authors consider that surgery with traditional fixed-angle devices (blade plate and DCS) is difficult and fracture fixation sometimes unstable [1]. Retrograde nailing is also an option for the treatment of distal femur fractures that might have superior outcomes compared to anatomic locking plate devices [14]. However, type C3 fractures with severe comminution may not be optimal for stabilization with a nail [15].

Clinical studies have generally shown good results with DCS plate devices (81% good or excellent results) [16]. A recent prospective multicenter randomized controlled trial comparing the less invasive stabilization system with the minimally invasive DCS System concluded that there was no advantage to the locking plate design in the management of distal femoral fractures [17]. The present study evaluated clinical and radiographic outcomes after open reduction internal fixation (ORIF) of distal femoral fractures with a DCS plating system. The results confirm our hypothesis that adequate fracture fixation and satisfactory functional outcome may be achieved with this device because it is readily available and suitable for severely comminuted with a very low fracture line.

The average time to radiological union in different series [3, 18] ranged from 8 to 22 months with an average of 12.6 weeks in our series. Incidence of non-union is reported from 0% to 19% with the use of locking plates [19]. In our series, delayed union was observed in nine cases (3.7%) and non union in six cases (2.5%). Implant failure ranges from 0 to 20% [19]. In the study of Hsu *et al*. there were six patients (13.6%) experiencing early failure of fixation with lateral locked plating which had been attributed to sagittal oblique fracture pattern, longer working length and post-operative sagittal malalignment. So they advised additional fixation such as anterior or medial plate to avoid catastrophic early failure in these cases [20]. We did not have any implant failure in our series.

Mean knee ROM ranged from 100° to 121° in studies using locking plates [3, 21] and in our study the mean ROM was 115.7°. Knee stiffness was the major complication encountered in the study of Sié *et al*. it was attributed to prolonged immobilization and delay in performing surgery and rehabilitation program due to socioeconomic and logistic reasons [10]. In the present study, we were able to initiate physical therapy and ROM exercises immediately after splint removal. At the final follow-up, the KSS and Lysholm Knee Score were correlated with excellent function and low disability. Mean KSS was comparable with other studies that utilized same scoring system for functional evaluation. Doshi et *al*. reported mean KSS score of 88.8 in 24 elderly patients with distal femur fractures fixed with locking plates inserted with minimally invasive plate osteosynthesis (MIPO) techniques [3].

Our study has several limitations. First and foremost, it´s retrospective nature and the biases inherent to such study designs. Furthermore, the majority of our patients had relatively high-energy trauma compared with other clinical trials. In addition, only one type of plating was tested, so no direct comparison may be made with other osteosynthesis methods (locking plates, retrograde nails).

## Conclusion

This retrospective study reports a single institution study, where all the cases were operated by experienced surgeons, using the same implant, same operative approach and uniform postoperative rehabilitation protocol. Our study conclusively establishes that excellent functional outcome can be achieved with ORIF using DCS plate inserted with skilled surgical technique in distal femur fractures including the ones that are comminuted or occurring in osteoporotic bone with a negligible complication rates.

### What is known about this topic

- The optimal management of distal femoral fractures is still controversial;
- While locked plating is widely prescribed in distal femoral fractures, DCS plating system is not frequently used because some authors consider that surgery with traditional fixed-angle devices (blade plate and DCS) is difficult and fracture fixation sometimes unstable.

### What this study adds

- A total number of 240 patients with distal femur fractures were managed with DCS plate system; the mean follow-up period was 33 months (26-62 months): clinico-radiological progression of fracture union as well as the functional outcome were studied;
- Our study conclusively establishes that excellent functional outcome can be achieved with ORIF using DCS plate inserted with skilled surgical technique in distal femur fractures including the ones that are comminuted or occurring in osteoporotic bone with a negligible complication rates.
